# High-Precision LCCD-Based Focus Metrology for I-Line Lithography: Multi-Sample Repeatability and Adaptability Evaluation

**DOI:** 10.3390/mi17060714

**Published:** 2026-06-11

**Authors:** Hengrui Guan, Xinxin Zhao, Yuheng Chu, Wuhao Liu, Yongxing Yang, Dapeng Kuang, Maoxin Song, Mingchun Ling, Jin Hong

**Affiliations:** 1 Graduate School of Science Island, University of Science and Technology of China, Hefei 230026, China; hengrui@mail.ustc.edu.cn (H.G.); heyuee@mail.ustc.edu.cn (X.Z.); chuyuheng@aiofm.ac.cn (Y.C.); liuwuhao@ustc.edu (W.L.); nameyyan@mail.ustc.edu.cn (Y.Y.); 2 Anhui Institute of Optics and Fine Mechanics, Hefei Institutes of Physical Science, Chinese Academy of Sciences, Hefei 230031, China; kuangdapeng@aiofm.ac.cn (D.K.); hongjin@aiofm.ac.cn (J.H.)

**Keywords:** LCCD-based focus metrology, local focus-height measurement, repeatability, material-surface adaptability, I-line inspection, semiconductor metrology

## Abstract

Achieving stable local focus-height measurement across different material surfaces is important for I-line-lithography-related inspection, where sub-micrometer height deviations can affect imaging quality, exposure uniformity, and subsequent autofocus performance. This study evaluates the local focus-height repeatability of a linear charge-coupled device (LCCD)-based focus metrology system under several I-line-lithography-related material-surface conditions. The prototype integrates fiber-coupled LED illumination, telecentric projection and imaging optics, reference marks, and a two-step localization procedure based on template matching and centroid estimation; the dual-wavelength source is treated as part of the fixed optical configuration. Tests were performed on silicon wafers, GaAs bright substrates, sapphire, infrared transmissive material, and SiC, covering different reflectivity levels and surface structures. The measured peak-to-valley repeatability was 35–37 nm for highly reflective samples and 40–54 nm for intermediate- or low-reflectivity and microstructured samples, all below the selected 70 nm conservative engineering criterion derived from the depth-of-focus estimate. These results indicate that the integrated LCCD measurement chain maintained stable local repeatability within the tested material-surface range, providing experimental support for further development of local focus metrology and precision optical inspection.

## 1. Introduction

In modern I-line lithography, precise focus control and wafer leveling are critical for imaging quality, exposure uniformity, and the effectiveness of subsequent autofocus and leveling operations. As process nodes advance to 350 nm and below, even micrometer- or sub-micrometer-scale deviations in wafer surface height can significantly impact lithography outcomes [1,2,3]. For this reason, the focus-sensing module must first provide a stable local height response, because the repeatability of local height measurements forms the basis for reliable focus estimation and correction. Therefore, focus metrology for I-line lithography should provide not only sufficient height-measurement sensitivity, but also stable local repeatability under different material-surface conditions, so that the focus-sensing result remains reliable in multi-sample inspection and process-related measurement scenarios [4,5]. Beyond conventional semiconductor exposure, I-line lithography is also used in lithography-related micro/nano fabrication and precision optical inspection, where stable local focus sensing remains important for pattern fidelity and subsequent alignment.

Traditional non-contact optical measurement methods, such as structured light projection and moiré fringe techniques, are widely used for surface profiling and height measurement [6,7,8,9,10]. Structured light can rapidly capture full-field surface information but offers limited spatial resolution at the micro- and nano-scale and is sensitive to surface optical properties [11,12,13]. Moiré fringe techniques can achieve higher measurement accuracy but are typically constrained by interference noise and measurement speed [14,15]. Overall, these methods often face instability or reduced precision when applied to multiple sample types, varied materials, or complex surface conditions.

In practical industrial applications, variations in optical properties across different materials—such as highly reflective silicon, mirror-like GaAs, and microstructured SiC—can significantly affect focus signals, increasing the difficulty of achieving consistent multi-sample measurements [16,17,18,19,20]. Recent studies on plasmonic and resonant micro/nano optical sensors have also shown that material composition, surface geometry, and localized optical-field or spectral response can strongly influence the detected optical signal [21,22]. Although these sensing mechanisms differ from the optical triangulation approach used in this work, they further indicate that material- and structure-dependent signal variations should be considered in precision optical measurement. For single-point localization at sub-pixel precision, image-based centroid interpolation algorithms are commonly employed. Centroid interpolation calculates the intensity-weighted center of image features, providing nanometer-level sensitivity under stable imaging conditions [23,24]. However, its performance can degrade under variations in signal intensity, reflectivity, or surface microstructure, highlighting the need for additional processing strategies to ensure consistent feature extraction across diverse samples [25,26,27,28,29].

Recent lithography-related focus and leveling metrology studies have reported nanometer- to sub-100-nanometer-level performance under different evaluation conditions. For example, a digital-grating focusing and leveling sensor achieved 10 nm measurement resolution and tens-of-nanometers accuracy under controlled conditions, while process patterns on the wafer surface were still shown to introduce material- or pattern-dependent measurement errors [3]. A structured-illumination wafer focus measurement system reported rapid defocus measurement with an error below 0.06 μm [4]. In addition, grating-shearing interferometry has demonstrated wafer focus measurement precision of approximately 30 nm [30]. These studies indicate that high-precision focus metrology has reached the nanometer scale, but the reported metrics are not directly equivalent because they are obtained using different sensing principles, sample conditions, and evaluation definitions. In this context, the present work focuses on a different but complementary question: whether an LCCD-based optical-triangulation measurement chain can maintain 10 nm vertical resolution and 35–54 nm peak-to-valley local repeatability when the tested material surface is changed under a fixed optical configuration and processing workflow.

In this work, the linear charge-coupled device (LCCD)-based focus metrology system is evaluated with emphasis on local peak-to-valley repeatability under sample-to-sample material and surface variations. The system integrates fiber-coupled LED illumination, telecentric projection and imaging optics, reference marks, and a two-step localization procedure. In this procedure, template matching is first used to identify the target regions, and centroid estimation is then applied to determine the sub-pixel positions of the measurement marks. The illumination wavelengths were kept fixed as part of the optical configuration and were not treated as independent experimental variables. Under the same optical layout, acquisition settings, stage motion sequence, and signal-processing workflow, repeated local height measurements were carried out on silicon wafers, GaAs bright substrates, sapphire, infrared transmissive material, and SiC. By comparing the resulting peak-to-valley repeatability, this study examines the measurement consistency of the same LCCD-based local focus metrology system under different material-surface conditions. Compared with studies that mainly characterize a focus sensor under a single reference surface, this work emphasizes whether the same LCCD-based local measurement chain can maintain peak-to-valley repeatability when the tested material surface is changed. The present work is therefore focused on local repeatability evaluation, while full-wafer leveling, photoresist-coated wafers, and multilayer thin-film stacks require further dedicated investigation.

## 2. System Principle and Experimental Setup

### 2.1. Measurement Principle

**Figure 1:**
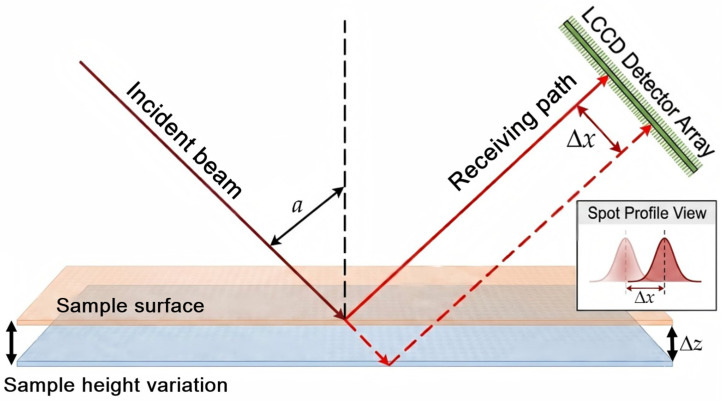
Basic Principle of Optical Triangulation.

In the proposed focus metrology approach, optical triangulation is used to convert minute variations in sample height into measurable lateral shifts on the LCCD detector [31,32,33,34,35]. As illustrated in Figure 1, when the sample surface moves along the measurement direction, the corresponding image feature shifts on the detector plane, allowing the focus offset to be determined from the detected signal displacement. Under constant system structural parameters, the relationship between the lateral displacement Δx on the detector and the sample height variation Δz is defined as:

(1) Δx=2Δzsinα⋅β,

where β is the lateral magnification of the LCCD calibration system, and α is the oblique incidence angle of the chief ray relative to the sample normal, equal to the exit angle in the imaging path. Equation (1) shows that, with fixed system parameters, sample height changes are converted into shifts in the detector feature position, enabling measurement of focus offset or height variations.

In this system, the LCCD acquires line-scan response signals, with sample height variations manifested as shifts in the feature position on the LCCD. Feature extraction and fine localization, combined with system calibration, enable high-sensitivity detection of minute height changes. Variations in material-surface conditions affect signal intensity, contrast, and distribution on the LCCD, influencing feature extraction stability. Repeatability under varying material-surface conditions is analyzed subsequently.

### 2.2. System Composition and Experimental Setup

**Figure 2:**
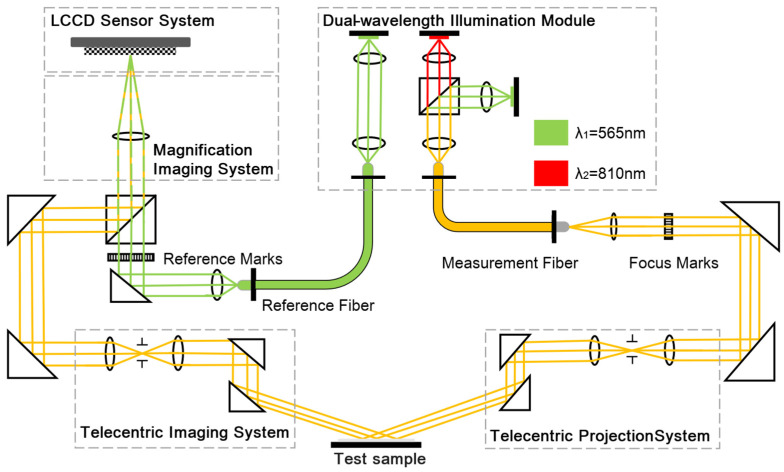
Overall structure of the LCCD-based local focus metrology system. The sample in the optical path represents the tested material surface rather than a specific photoresist-coated wafer.

The experimental setup, shown in Figure 2, consists of an illumination module, measurement optical path, reference optical path, telecentric projection and imaging systems, magnification system, LCCD detector, and data acquisition/processing modules. The measurement light, delivered by the illumination module, is projected onto the sample surface. The reflected signal is collected and magnified before being captured by the LCCD, producing a line-scan signal for measurement computation.

The measurement system employs a dual-wavelength LED illumination scheme, consisting of 565 nm light with a bandwidth of 103 nm and 810 nm light with a bandwidth of 30 nm. This wavelength pair was selected within the 500–850 nm metrology band specified in the system design requirements and is used as a sensing illumination configuration rather than as a lithographic exposure condition. The 565 nm component lies in the visible band and provides sufficient LCCD response and signal contrast for centroid-based localization, while the 810 nm component introduces a near-infrared contribution to provide an additional optical response for sample surfaces with different reflectivity or scattering characteristics. The selected wavelengths also fall within the effective spectral response range of the LCCD detector, with representative quantum efficiencies of approximately 0.76 at 500–600 nm and 0.65 near 800 nm according to the detector specifications. The finite spectral bandwidth of the LED sources can also reduce the sensitivity to coherence-related artifacts compared with narrow-linewidth illumination. In this work, the 565/810 nm combination was kept fixed to obtain stable and comparable LCCD signals across the tested material surfaces, and wavelength selection itself was not treated as an independent optimization variable.

After being coupled into optical fibers, the illumination is transmitted through dedicated optical components before entering the projection system. Both the projection and imaging ends adopt dual-telecentric configurations with an oblique incidence angle of 81.5°, which helps minimize geometric distortion and ensures a consistent mapping between local height variations in the tested sample and detector signal shifts. The reference optical path delivers a stable dual-peak signal at the detection end, providing a reliable baseline for measurement comparison across multiple samples.

Signals reflected from the sample surface are collected via the telecentric imaging system and relayed to the calibration magnification path, which has a magnification factor of 14.5. This magnification enlarges the feature images on the LCCD, forming clear, resolvable distributions that facilitate precise feature extraction and sub-pixel localization. Detection relies on the stability of these feature positions rather than individual pixels, so imaging fidelity directly impacts the reproducibility of measurement outputs.

The LCCD detection module comprises 2048 pixels, each 14 μm in size, and converts the captured line-scan signals into digital data for processing. For repeatability evaluation, a nano-positioning stage introduces controlled micro-height variations, simulating single-point measurements. Across all sample tests, illumination, system alignment, acquisition parameters, and stage motion protocols are strictly maintained constant. These system parameters and configurations are summarized in Table 1, providing a comprehensive reference for the optical setup and ensuring consistent measurement conditions for evaluating repeatability across different material-surface conditions.

Since the subsequent localization procedure relies on the structural features of the measurement and reference marks, the main geometric parameters of the mark module were specified before signal extraction. The measurement marks consist of five unequally spaced slit-like features, which generate the central multi-peak signal on the LCCD. The reference marks are arranged on both sides of the measurement signal and form two dual-peak reference signals, providing a stable positional baseline for relative-displacement calculation. Table 2 summarizes the key geometric parameters of the mark module, including the physical mark size, mark arrangement, reference-line spacing, reference-group separation, and the geometric projected widths on the LCCD. These parameters define the spatial basis for template matching, centroid localization, and relative-displacement calculation.

### 2.3. Signal Processing and Measurement Procedure

After signal acquisition is completed, the LCCD outputs raw data in the form of a line-scan response distribution across the detection plane. In this system, the detector signal consists primarily of two components: reference marks and measurement marks. The reference marks provide a stable measurement baseline, while the measurement marks encode the sample height variations. The subsequent measurement extraction does not rely on the absolute position of a single peak; instead, it is based on the relative positional relationship between the reference and measurement marks.

**Figure 3:**
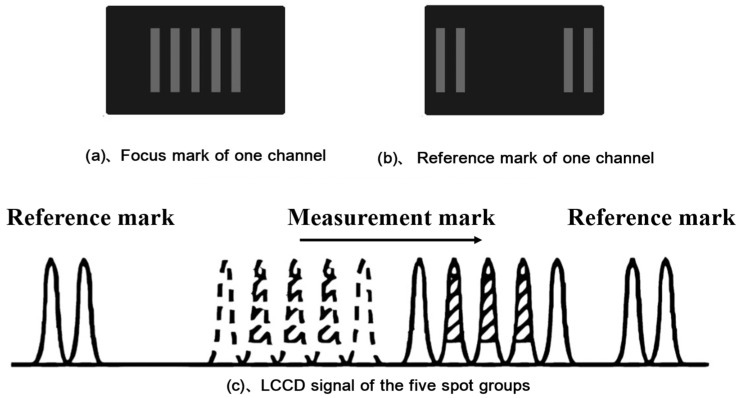
Measurement and reference marks, and their corresponding LCCD line-scan signals for the five-spot groups. (**a**) Schematic representation of the measurement focus marks for one channel, indicating the positions used to capture sample height variations for subsequent LCCD signal analysis. (**b**) Illustration of the reference marks for one channel, providing stable positional features used as a baseline to evaluate relative displacements in the measurement process. (**c**) Line-scan output of the LCCD showing the relative positions of the measurement marks as central multi-peak signals between two reference marks, corresponding to the five-spot groups. These signals form the basis for feature extraction and sub-pixel localization to quantify single-point repeatability.

Figure 3 illustrates the measurement and reference marks, along with their corresponding LCCD line-scan signals for the five-spot groups. The reference marks form two relatively stable dual-peak signals located on either side of the measurement signal, while the measurement marks generate a multi-peak response in the central region. Sample height variations are reflected as positional shifts in the central measurement marks relative to the reference marks on both sides. Therefore, the system output fundamentally represents the relative positional changes between the reference and measurement marks, and the evaluation of repeatability is conducted based on fluctuations in this relative position.

**Figure 4:**
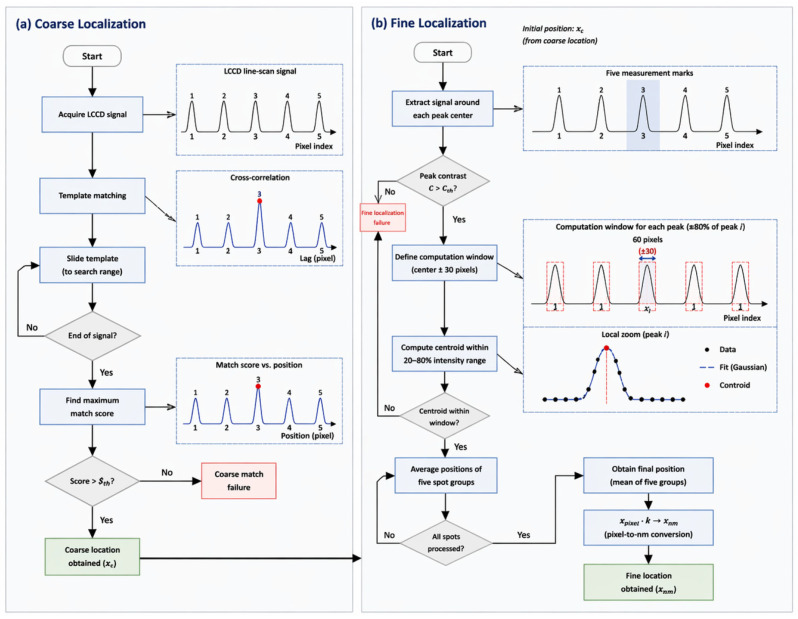
Flowchart of the two-step image processing algorithm for LCCD signal analysis. (**a**) Coarse localization step where the LCCD line-scan signal is acquired, template matching is performed, and the initial measurement target regions are identified. A coarse location is obtained when the maximum match score exceeds the threshold, providing a reliable initial range for subsequent fine localization. (**b**) Fine localization step using centroid-based sub-pixel estimation within the previously identified target regions. Signals are extracted, validated for peak contrast, and the centroid of each measurement mark is computed. The final positions are averaged across the five-spot groups and converted to nanometers to quantify single-point displacement with high precision.

Considering that the intensity, contrast, and peak profiles of the LCCD response signals may vary with different material-surface conditions, a two-step image processing algorithm was used to extract stable feature positions from the detected line-scan signals, as illustrated in Figure 4. The first step is template-based coarse matching. In this stage, the known structural features of the reference and measurement marks are used to search the entire line-scan signal and identify the corresponding target regions. This process provides approximate position ranges for both the reference marks and measurement marks. The role of coarse matching is not to determine the final high-precision positions, but to ensure that the same measurement targets can be consistently and correctly located under different material-surface conditions and measurement cycles, thereby providing reliable initial regions for subsequent fine localization.

Following coarse matching, centroid-based fine localization is performed within the identified target regions to determine the feature positions with sub-pixel precision. Unlike the coarse matching step, which mainly provides approximate target locations, centroid localization calculates the intensity-weighted center of each extracted signal peak by using the grayscale distribution within the local region. This allows the feature center to be estimated beyond the physical pixel pitch of the LCCD and helps reduce the influence of pixel discretization, local peak-shape variations, and random noise on the position result. Therefore, the feature positions used for displacement calculation in this system are determined from the centroid localization results.

The final measurement output is defined as the relative positional change between the measurement marks and the reference marks, rather than the absolute position of a single signal peak. This relative-position definition helps reduce the influence of common positional drift and provides a consistent basis for comparing measurements across different samples. Repeatability is then evaluated from the fluctuation range of the measurement outputs obtained from multiple independent measurements. To ensure experimental comparability, all sample tests were processed using the same template-matching procedure, centroid localization method, and output definition.

After the target regions are identified by template matching, the sub-pixel position of each signal peak is determined by centroid localization. For the k-th extracted peak, the centroid position $x_{k}$ is calculated within the local computation window $W_{k}$ as:

(2) $$x_{k}=\frac{\sum_{p\in W_{k}}p\left[I\left(p\right)-I_{b}\right]}{\sum_{p\in W_{k}}\left[I\left(p\right)-I_{b}\right]},$$

where p is the pixel index, $I\left(p\right)$ is the LCCD gray-level intensity, and $I_{b}$ is the local background or threshold baseline used for centroid calculation. The position of the measurement signal is then obtained by averaging the five measurement marks:

(3) $$x_{m}=\frac{1}{5}\sum_{k=1}^{5}x_{m,k}.$$

For the reference signal, the two peaks in each reference group are first averaged to obtain the left and right reference-group centers:

(4) $$x_{r,L}=\frac{x_{r,L1}+x_{r,L2}}{2},x_{r,R}=\frac{x_{r,R1}+x_{r,R2}}{2}.$$

The reference position is then defined as the average of the two reference-group centers:

(5) $$x_{r}=\frac{x_{r,L}+x_{r,R}}{2}.$$

Therefore, the relative pixel displacement used for height calculation is expressed as:

(6) $$\Delta x_{rel}=\left(x_{m}-x_{r}\right)-\left(x_{m,0}-x_{r,0}\right),$$

where $x_{m,0}$ and $x_{r,0}$ denote the measurement-mark and reference-mark positions at the initial reference state. According to the optical triangulation relationship in Equation (1), the corresponding local height displacement is calculated as:

(7) $$\Delta z=\frac{s_{p}\Delta x_{rel}}{2\beta \sin\alpha },$$

where $s_{p}$ is the LCCD pixel size, β is the magnification factor used for height conversion, and α is the incidence angle relative to the sample normal. In this way, the final height output is determined from the relative displacement between the averaged measurement marks and the reference marks, rather than from the absolute position of a single signal peak.

With the optical layout, mark geometry, signal extraction procedure, and relative-output definition established, the next step was to examine whether the same measurement chain could maintain consistent repeatability when the material and surface condition of the tested sample changed. In the present system, the local height output is calculated from the relative displacement between the averaged measurement marks and the reference marks, and the same template-matching and centroid-localization workflow is used for all samples. Therefore, the subsequent experiments were designed to keep the optical alignment, illumination setting, acquisition parameters, stage motion sequence, and processing procedure unchanged while replacing only the sample type. The following section describes the selected I-line-lithography-related material samples, the experimental configuration, and the repeatability test procedure used to compare the local measurement response under different material-surface conditions.

## 3. Experimental Design and Evaluation Methodology

### 3.1. Tested Material Samples

To evaluate the local repeatability of the LCCD-based focus metrology system under different material-surface conditions, five material samples related to I-line lithography and precision optical inspection were selected: silicon wafer, GaAs bright substrate, sapphire, infrared transmissive material, and SiC. These samples were used to cover typical differences in surface reflectivity, optical contrast, and local surface structure that may influence the LCCD response signal. In this way, the system repeatability could be compared under different material-surface responses, rather than being assessed only on a single reference wafer.

The silicon wafer was used as the reference sample because of its smooth polished surface and relatively stable reflective properties, providing a baseline for evaluating the intrinsic repeatability of the system under well-controlled optical conditions. The GaAs bright substrate has a mirror-like surface and high reflectivity, making it suitable for assessing the system response on highly reflective samples. Sapphire, with a smooth surface and intermediate optical characteristics, further extends the tested material range between highly reflective and low-reflectivity samples. In contrast, the SiC and infrared transmissive material samples represent less favorable optical conditions, including lower reflected signal intensity, scattering, and local surface-structure effects, which may influence the contrast and peak profile of the LCCD signal. Therefore, the selected samples were intended to cover typical material-surface responses relevant to the repeatability evaluation, rather than to reproduce a complete photoresist-coated wafer stack. The effects of photoresist layers and multilayer thin-film structures are left for further dedicated investigation.

To ensure that the repeatability comparison mainly reflected the influence of material and surface characteristics, all samples were prepared within the measurable range of the system and mounted under the same optical alignment and acquisition conditions. For each sample, the repeatability test consisted of 20 stable-position measurements, including ten measurements at the high position and ten measurements at the low position. The same illumination parameters, LCCD acquisition settings, motion protocol, and signal processing workflow were used throughout the tests. This arrangement minimized the influence of system configuration and data-processing differences, allowing the measured variations to be compared on a consistent experimental basis. The detailed specifications of the experimental samples are summarized in Table 3.

### 3.2. Experimental Setup and System Configuration

**Figure 5:**
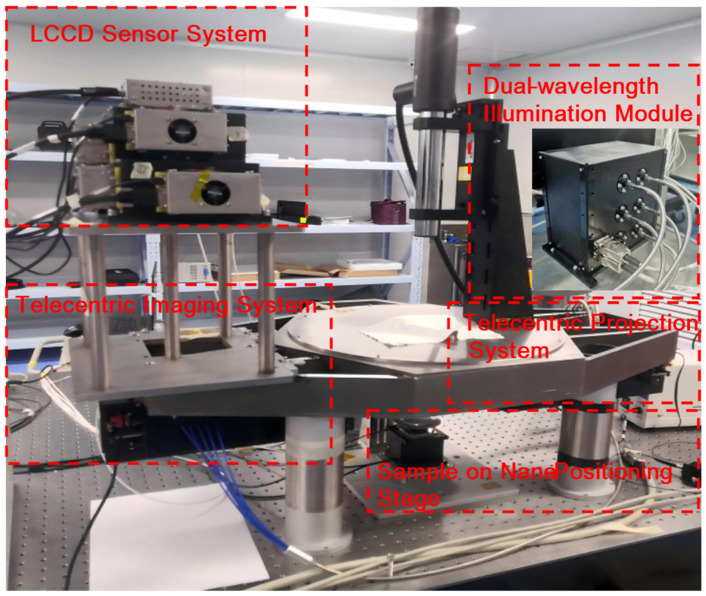
The experimental setup of the LCCD-based focus metrology system.

Figure 5 illustrates the experimental setup of the LCCD-based focus metrology system, including the sample positioning stage and the layout of the optical projection and imaging components. To ensure the reliability and comparability of the measurements, all samples were tested under uniform experimental conditions. The laboratory temperature was maintained at approximately 22 ± 1 °C, and environmental light interference was minimized using an optical table and surrounding light shielding, while efforts were made to reduce vibrations affecting the system, ensuring stable LCCD signal acquisition.

The samples were mounted on a nano-positioning stage, which provided precise displacement along the optical axis. The motion range was set to ensure that the measurement-mark response on the LCCD remained within the effective acquisition region. During data collection, the LCCD exposure time and sampling frequency were kept unchanged for all samples. The acquired line-scan signals were then processed using the same two-step workflow. Template-based coarse matching was first used to locate the reference and measurement-mark regions, followed by centroid localization to determine their sub-pixel feature positions.

Furthermore, the system was calibrated to establish the conversion relationship between pixel displacement and actual height displacement, ensuring that the output displacement results are consistent and comparable. Throughout the experiments, the same system settings and processing workflow were used for all samples, allowing the repeatability results to be compared under a unified measurement condition. The auxiliary equipment used during the experiments is summarized in Table 4.

### 3.3. Repeatability Test Procedure and Evaluation Metric

**Figure 6:**
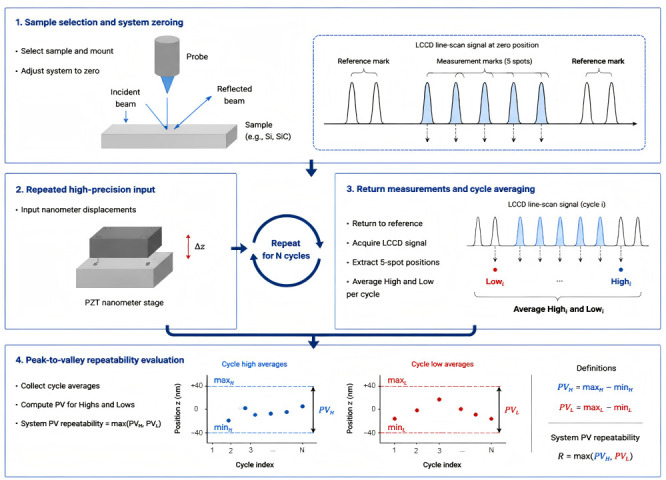
Flowchart of the peak-to-valley repeatability test procedure based on repeated high- and low-position measurements.

To evaluate the repeated measurement performance of the LCCD-based focus metrology system under different material-surface conditions, a peak-to-valley repeatability test was carried out following the procedure shown in Figure 6 [36,37]. The test was designed to characterize the local focus-height repeatability at a fixed measurement position, rather than to reconstruct full-wafer topography or separate global wafer tilt, wafer bow, and local surface-shape variation. Before testing, each sample was mounted on the nano-positioning stage, and the system was adjusted to the same reference state. This step was used to keep the initial height baseline comparable when different samples were replaced, so that the subsequent repeatability results could be compared under a consistent local measurement condition.

During the test, the nano-positioning stage repeatedly moved between the high and low height positions along the measurement direction. After each movement, the LCCD line-scan signal was collected, and the positions of the five measurement marks were extracted using the same template-matching and centroid-localization procedure. For each cycle, the stable output segments corresponding to the high and low positions were averaged to obtain the cycle-level height outputs, as defined in Equation (8):

(8) $${\bar{z}}_{H,i}=\frac{1}{M}\sum_{j=1}^{M}z_{H,i,j},\;{\bar{z}}_{L,i}=\frac{1}{M}\sum_{j=1}^{M}z_{L,i,j},$$

where ${\bar{z}}_{H,i}$ and ${\bar{z}}_{L,i}$ are the averaged height outputs of the high and low positions in the i-th measurement cycle, respectively. $z_{H,i.j}$ and $z_{L,i.j}$ denote the sampled height values within the corresponding stable segments, and M is the number of sampled points used for averaging.

The peak-to-valley variations at the two height positions were then calculated separately, as shown in Equation (9):

(9) $$PV_{H}=\max\left({\bar{z}}_{H,i}\right)-\min\left({\bar{z}}_{H,i}\right),\;PV_{L}=\max\left({\bar{z}}_{L,i}\right)-\min\left({\bar{z}}_{L,i}\right),$$

where $PV_{H}$ and $PV_{L}$ represent the peak-to-valley variations in the high-position and low-position outputs, respectively. Since the return error may appear at either height position, the larger of these two values was used as the final peak-to-valley repeatability for each sample, as expressed in Equation (10):

(10) $$R_{PV}=\max\left(PV_{H},PV_{L}\right),$$

Here, $R_{PV}$ describes the maximum variation observed during repeated high- and low-position measurements. For all tested samples, the illumination setting, LCCD acquisition parameters, stage motion sequence, and data-processing method were kept unchanged. Therefore, the obtained $R_{PV}$ values were used to compare the influence of different material-surface conditions on the measurement repeatability.

## 4. Experimental Results and Analysis

### 4.1. System Performance Validation

Before evaluating repeatability under different material-surface conditions, the baseline local measurement performance of the LCCD-based focus metrology system was first characterized using a bare silicon wafer. This step was necessary to establish a stable baseline before evaluating the additional variations associated with different material reflectivity and surface structures. Specifically, linearity, vertical resolution, and short-term stability were tested to confirm whether the system could provide reliable height response and stable output under controlled optical conditions. The bare silicon wafer was selected for this baseline validation because of its smooth surface and relatively stable reflective properties, which help minimize sample-dependent effects during fundamental performance testing.

**Figure 7:**
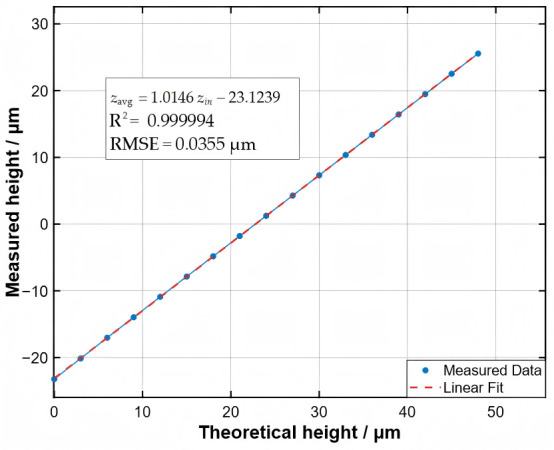
Linear relationship between the theoretical input height and the averaged measured height output of the proposed system.

The system linearity was evaluated using the nano-positioning stage, as shown in Figure 7. Before calibration, the same bare silicon wafer was mounted on the stage, and the system was adjusted to the reference state. The initial stage position and the corresponding system output were recorded as the zero-displacement reference. The nano-positioning stage was then driven along the measurement direction from 0 μm to 50 μm with a step interval of 2 μm. At each displacement position, the LCCD height output was recorded after the stage reached a stable state. The collected input–output data were fitted using the least-squares method to establish the calibration relationship between the nominal stage displacement and the measured height output. The fitting result gave a correlation coefficient of R^2^ = 0.999994 and a root mean square fitting error of 35.5 nm. The fitted intercept corresponds to the height offset of the selected optical reference state and does not affect the evaluation of the linear response. The measured points closely followed the fitted straight line over the tested range, indicating that the calibrated system output responded consistently to the applied height changes. This result provides the calibration basis for the subsequent repeatability evaluation, in which height variations from different samples were compared under the same calibration condition. During this calibration, the nominal displacement provided by the nano-positioning stage was used as the displacement reference, and the same height-conversion factor obtained from the bare-silicon calibration was kept fixed for all subsequent sample-repeatability tests.

**Figure 8:**
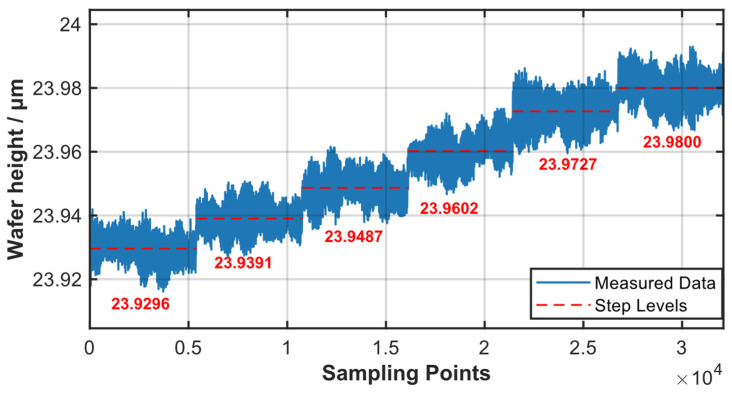
Measured system response for vertical resolution characterization.

As shown in Figure 8, the system resolution was evaluated using the nano-positioning stage and a bare silicon wafer. Stepwise height inputs of 20 nm, 10 nm, and 5 nm were applied within a fixed measurement range, and the corresponding height sequences were recorded from the LCCD output. The 20 nm and 10 nm inputs produced distinguishable step responses, while the 5 nm input was not consistently separated from the short-term fluctuation. Based on this comparison, the effective system resolution was determined to be 10 nm under the tested condition. This resolution test provided a baseline for interpreting the subsequent repeatability measurements.

**Figure 9:**
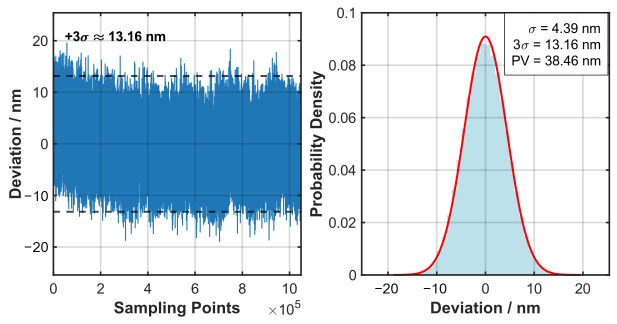
Stability evaluation of the experimental system over 1 × 10^6^ sampling points.

The system stability was evaluated under a fixed-height condition, as shown in Figure 9. The LCCD output signals were continuously collected for five minutes, and the height data were extracted using the same template-based coarse matching and centroid localization procedure. The three-sigma value of the single-point height fluctuation was 13.16 nm, and the peak-to-valley range was 38.46 nm. These results show that the short-term output fluctuation remained at the nanometer level under the tested condition, providing a baseline for the following multi-sample repeatability evaluation.

In summary, the baseline tests on the bare silicon wafer provided the reference performance of the LCCD-based focus metrology system under controlled material-surface conditions. The linearity test showed that the calibrated system output followed the applied height displacement over the tested range. The resolution test gave an effective system resolution of 10 nm, and the five-minute stability test showed a three-sigma fluctuation of 13.16 nm with a peak-to-valley range of 38.46 nm. These results indicate that the system response was sufficiently stable under the reference silicon-wafer condition to support the subsequent comparison of peak-to-valley repeatability across different material-surface conditions.

### 4.2. Multi-Sample Peak-to-Valley Repeatability Evaluation

After confirming the baseline local measurement performance of the LCCD-based focus metrology system, its peak-to-valley repeatability was further evaluated under different material-surface conditions. In lithographic focusing, the allowable height fluctuation is closely related to the depth of focus of the projection lens. For an I-line projection lens, the depth of focus can be approximately estimated by Equation (11):

(11) $$DOF=k_{2}\frac{\lambda }{NA^{2}},$$

where λ is the exposure wavelength, NA is the numerical aperture of the projection lens, and $k_{2}$ is a process-dependent coefficient. Recent I-line stepper specifications report 365 nm exposure wavelength and a numerical aperture of 0.45 for a 350 nm-class I-line exposure system [38]. Using these parameters and taking $k_{2}=0.4$ as a conservative process coefficient, the estimated depth of focus is approximately 0.72 μm. Therefore, one-tenth of this value, approximately 70 nm, was adopted as a conservative engineering criterion for repeatability evaluation, rather than as a universal lithographic acceptance limit. Since focus-sensor repeatability accounts for only one part of the overall focus-control error budget, this value provides a margin for other contributors, such as stage positioning error, leveling control error, thermal drift, and wafer-surface variation. The criterion was therefore used to examine whether the local measurement fluctuation of the focus sensor remained sufficiently smaller than the available focusing tolerance. Based on this criterion, the peak-to-valley repeatability of the system was tested on silicon wafers, GaAs bright substrates, sapphire, infrared transmissive material, and SiC.

**Figure 10:**
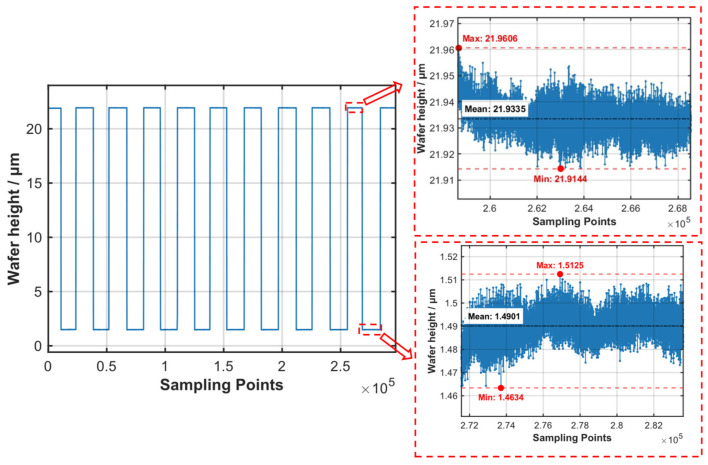
Silicon-wafer peak-to-valley repeatability evaluation based on repeated height measurements.

The initial repeatability tests were conducted on the bare silicon wafer. As a system baseline, the measurement curve for the silicon wafer is shown in Figure 10. Throughout repeated switching, the two height positions remained clearly distinguishable, and no significant decline in return consistency was observed. To quantify this behavior, the average value of the stable segment in each cycle was extracted, and the high and low mean values are listed in Table 5. Based on these cycle-level averages, the peak-to-valley values were 32 nm at the high position and 35 nm at the low position. The larger value, 35 nm, was therefore taken as the peak-to-valley repeatability of the system for the silicon wafer sample.

**Figure 11:**
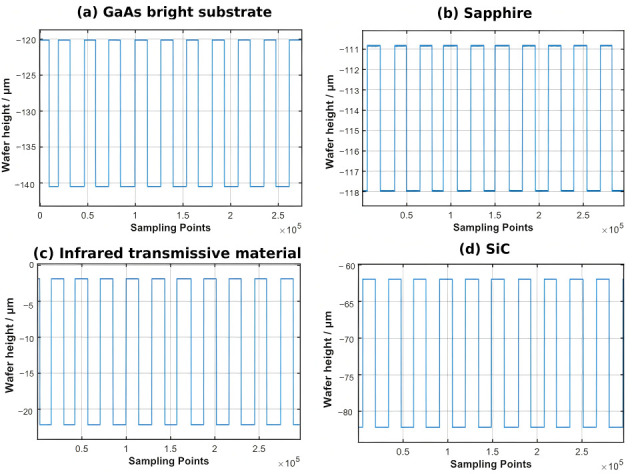
Repeated high- and low-position height measurement curves for four representative samples. (**a**) GaAs bright substrate; (**b**) Sapphire; (**c**) Infrared transmissive material; (**d**) SiC.

Following this, peak-to-valley repeatability experiments were conducted on four additional representative samples: GaAs bright substrate, sapphire, infrared transmissive material, and SiC. The experimental conditions were kept identical to those used for the bare silicon wafer, including the optical alignment, illumination setting, LCCD acquisition parameters, stage motion sequence, and signal-processing procedure. As shown in Figure 11, all four samples exhibited clear high- and low-position switching responses during repeated measurements, indicating that the same test procedure could be applied to these sample surfaces. For each sample, the stable output segments were averaged cycle by cycle using the same method as that used for the silicon wafer. The corresponding high-position variation, low-position variation, and final peak-to-valley repeatability, expressed as PV_H_, PV_L_, and R_PV_, are summarized in Table 6.

**Figure 12:**
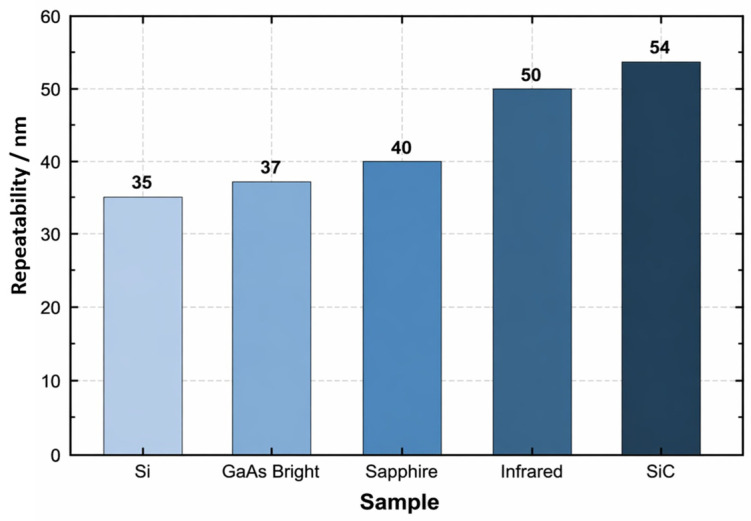
Peak-to-valley repeatability of LCCD focus measurements across different samples.

To compare the repeatability performance across different material-surface conditions, Figure 12 summarizes the peak-to-valley repeatability results for the five tested samples. The silicon wafer and GaAs bright substrate showed the smallest $R_{PV}$ values, 35 nm and 37 nm, respectively. Sapphire exhibited a slightly higher value of 40 nm, while the infrared transmissive material and SiC samples showed larger values of 50 nm and 54 nm. Because the optical layout, illumination setting, acquisition parameters, stage motion sequence, and signal-processing procedure were kept unchanged throughout the tests, the observed differences mainly reflect how the same local measurement chain responded to the tested material-surface conditions.

This trend is consistent with the surface and optical characteristics of the tested samples. The silicon wafer and GaAs bright substrate both have smooth and highly reflective surfaces, which are favorable for obtaining stable and well-defined LCCD peak features. Sapphire has a smooth surface but an intermediate optical response, and its repeatability result falls between those of the highly reflective samples and the less favorable samples. For the infrared transmissive material, part of the incident light may be transmitted rather than reflected back into the imaging path, reducing the effective reflected signal level. For SiC, the microstructured surface may introduce local scattering or peak-profile variation. These effects may increase the fluctuation of the extracted centroid positions, which is consistent with the larger $R_{PV}$ values observed for these two samples.

The present comparison does not quantitatively separate the individual contributions of reflectivity, scattering, surface microstructure, and peak-profile variation. Instead, it provides a repeatability-based evaluation of whether the same LCCD-based local measurement chain can maintain stable output under different material-surface conditions.

In summary, the same LCCD measurement configuration and localization procedure kept $R_{PV}$ below the selected 70 nm conservative engineering criterion for all five tested samples. The larger fluctuations observed for the infrared transmissive material and SiC are consistent with less favorable material-surface responses, such as reduced reflected signal level, local scattering, or peak-profile variation. These results provide an experimental comparison of the local measurement response under the tested material-surface conditions, while a quantitative decomposition of the individual optical factors remains a subject for further study.

It should also be clarified that the present evaluation focuses on repeatability rather than absolute accuracy. The reported $R_{PV}$ values describe the fluctuation range of repeated local height outputs under the same optical configuration and processing workflow. They do not fully quantify material-dependent systematic offsets or calibration-slope changes. Such effects may arise when the sample reflectivity, scattering behavior, or apparent optical reflection plane changes with material or surface condition. Therefore, the present results demonstrate local repeatability consistency within the tested material-surface range, while absolute accuracy across different material stacks requires further evaluation using traceable reference surfaces or calibrated coated-wafer samples.

## 5. Conclusions

This study evaluated the peak-to-valley repeatability of an LCCD-based local focus metrology system under several I-line-lithography-related material-surface conditions. Baseline tests on a bare silicon wafer first confirmed the linear height response, 10 nm vertical resolution, and short-term stability of the system under controlled measurement conditions. Multi-sample tests were then performed using the same optical configuration, acquisition settings, stage motion sequence, and localization procedure.

The measured $R_{PV}$ values were 35 nm for the silicon wafer, 37 nm for the GaAs bright substrate, 40 nm for sapphire, 50 nm for the infrared transmissive material sample, and 54 nm for SiC. All values were below the selected 70 nm conservative engineering criterion derived from the depth-of-focus estimate. The results also suggest a material-dependent trend: the smooth and highly reflective samples produced smaller repeated-position variations, whereas the infrared transmissive material sample and SiC showed larger fluctuations. This trend is consistent with the expected influence of sample-dependent optical response on centroid localization, although the individual effects of reflectivity, scattering, and local peak-profile variation were not separated quantitatively in this study.

Overall, the results indicate that the same LCCD measurement configuration and localization procedure can maintain local peak-to-valley repeatability within the selected 70 nm conservative engineering criterion for the tested material-surface range. These findings support the feasibility of using the proposed system for local focus-height repeatability evaluation under I-line-lithography-related material-surface conditions. It should be noted that the present study focuses on local repeatability rather than full-wafer leveling accuracy or complete coated-wafer focus response. Future work will further examine photoresist-coated wafers, multilayer thin-film stacks, material-dependent systematic offsets, and multi-point wafer-plane evaluation.

## Figures and Tables

**Table 1:** Summary of the main system parameters.

Category	Parameter Name	Value/Description
Measurement Principle	Measurement Method	Optical Triangulation
	Detection Mode	Line-scan detection based on LCCD
Illumination Module	Illumination Mode	Fiber-coupled illumination
	Illumination Wavelength	565 nm (bandwidth 103 nm) + 810 nm (bandwidth 30 nm)
Dual-Telecentric Optical Path Module	Projection and Imaging System Type	Double-telecentric optical path
	Chief-ray incidence angle relative to the sample normal, α	81.5°
Calibration Magnification Path Module	Imaging System Type	Telecentric imaging system
	Magnification System β	14.5
Mark Module	Measurement Mark Type	Five-spot measurement mark
	Reference Mark Type	Dual-peak reference mark
Detection Module	Detector Type	LCCD
	Detector model	Hamamatsu S11156-2048-01
	Number of Pixels	2048
	Pixel Size	14 μm

**Table 2:** The main geometric parameters of the mark module.

Category	Parameter	Value/Description
Measurement Marks	Number of measurement marks	5
	Physical mark width	0.1 mm
	Physical mark length	1.2 mm
	Slit arrangement	Unequally spaced
	Total width of five-mark group	4.5 mm
	Projected peak width on LCCD	≈15 pixels, geometric estimate
	Measurement group width on LCCD	≈689 pixels, geometric estimate
Reference Marks	Number of reference peaks	4
	Physical reference-line width	0.015 ± 0.001 mm
	Gap between two reference lines	0.06 ± 0.001 mm
	Reference-group separation	1.86 ± 0.001 mm
	Projected reference peak width on LCCD	≈16 pixels, geometric estimate
Signal Processing	Computation window width for each peak	60 pixels
	Pixel size of LCCD	14 μm
	Magnification factor used for height conversion	14.5

Note: The projected widths on the LCCD are geometric estimates calculated from the projected mark dimensions, the magnification factor of 14.5, and the LCCD pixel size of 14 μm. The actual signal peak width may vary with the imaging condition, optical point-spread function, and acquisition settings.

**Table 3:** The detailed specifications of these experimental samples.

Sample Type	Material/Sample Condition	Dimensions	Remarks
Silicon wafer	Polished silicon wafer	25 mm × 25 mm	Polished surface
GaAs bright substrate	Bare bright GaAs substrate	25 mm × 25 mm	Mirror-like high-reflectivity surface
SiC	Microstructured SiC sample	20 mm × 20 mm	Microstructured surface
Infrared transmissive material	Infrared transmissive glass	20 mm × 20 mm	Smooth low-reflectivity surface
Sapphire	Sapphire wafer	20 mm × 20 mm	Smooth surface

**Table 4:** Auxiliary equipment used for the experiments and their main specifications.

NO.	Name	Model	Main Parameters	Function/Test Item
1	Manual Tilt Stage	P520TS	Travel range 1.8 mrad, repeatability over full range ±0.015%, linearity over full range 0.12%	Align sample surface
2	Nano-Positioning Stage	P620.ZCD	Travel 50 μm, repeatability ±0.02%, linearity ±0.02%	Repeatability, linearity, stability test
3	Manual Lifting Stage	5100006	Travel 5 mm, repeatability ±0.2 μm, linearity 0.12%	Relative height range adjustment
4	Capacitive Sensor	D-E20.200	Measurement range 200 μm, linearity 0.02%	Sample height monitoring
5	Vibration Isolation Platform	ZTP24-12	1.2 × 2.4 m, load ≥ 800 kg, isolation ≥ 90%	Maintain vibration-isolated conditions
6	Vibration Sensor	731A-P31	Frequency range 0.05 Hz–450 Hz, sensitivity 10, 100, 1000 V/g, measurement range 0–5 g	Monitor vibration conditions
7	Temperature Sensor	P0.251512, H3L2000	Range 50–250 °C, accuracy ±0.1 °C	Confirm temperature conditions

**Table 5:** Cycle-level height outputs used for silicon-wafer peak-to-valley repeatability evaluation.

Cycle No.	Mean Value of High Position (μm)	Mean Value of Low Position (μm)
1	21.9357	1.4692
2	21.9535	1.4854
3	21.9637	1.4891
4	21.9331	1.4888
5	21.9340	1.4877
6	21.9418	1.5042
7	21.9317	1.4873
8	21.9521	1.4965
9	21.9340	1.4734
10	21.9335	1.4901

**Table 6:** Peak-to-valley repeatability results for the five representative samples.

Sample	Surface Condition	PV_H_ (nm)	PV_L_ (nm)	R_PV_ (nm)
Silicon wafer	Polished smooth surface	32	35	35
GaAs bright substrate	Mirror-like high-reflectivity surface	37	29	37
Sapphire	Smooth intermediate-reflectivity surface	40	38	40
Infrared transmissive material	Smooth low-reflectivity surface	44	50	50
SiC	Microstructured surface	51	54	54

## Data Availability

The data underlying the results presented in this paper are not publicly available at this time but may be obtained from the authors upon reasonable request.
